# Editorial: Bridging the gap before and after birth: methods and technologies to explore the functional neural development in humans

**DOI:** 10.3389/fnhum.2015.00571

**Published:** 2015-10-14

**Authors:** Marika Berchicci, Silvia Comani

**Affiliations:** ^1^BIND - Behavioral Imaging and Neural Dynamics Center, University “G. d'Annunzio” of Chieti-PescaraChieti, Italy; ^2^Department of Human Movement, Social and Health Sciences, University of Rome “Foro Italico,”Rome, Italy; ^3^Department of Neuroscience, Imaging and Clinical Sciences, University “G. d'Annunzio” of Chieti-PescaraChieti, Italy; ^4^Casa di Cura Privata Villa SerenaCittà Sant'Angelo, Italy

**Keywords:** fetus, infant, neuroimaging, brain development, autonomous nervous system, fetal heart rate

Early human development from late gestation to the neonatal period is a critical time in the individual's life span. Preterm birth or medical issues affecting the brain function during late gestation or the first months of life may lead to an avalanche of neurodevelopmental problems, including cognitive deficits and motor disability, with lifelong consequences for the individuals, their families, the health care system and the society (de Kieviet et al., 2012; March of Dimes, PMNCH, Save the Children, WHO, 2012). Despite the need for the alleviation of perinatal adversities, effective monitoring methods and clinical diagnostic procedures able to reduce fetal impairment and advance neonatal and infant healthcare are still missing.

The focus of this E-Book is on the most recent developments and findings in the field of non-invasive monitoring of the central and autonomous nervous systems in fetuses and infants. The contributed opinion, review and original research articles included in this E-Book cover different methodological, clinical, functional, and structural topics, with the purpose to disseminate the knowledge on novel perinatal diagnostic tools and procedures, and to share the findings of high quality functional assessment of the developing human brain, and of clinical studies on pathological neurodevelopment.

The fundamental requirement for a healthy functional development of the brain is the maintenance of an adequate blood flow and oxygenation. Inadequate brain perfusion is the main cause of preterm brain damage, which can be diagnosed by high-frequency ultrasound. Camfferman et al. (2015) performed an *in vitro* experiment using a microvessel flow phantom designed to mimic preterm cerebral perfusion and assess blood flow velocity. Their flow phantom allowed the visualization of the vessels, but flow velocity and vessel diameter were overestimated. Therefore, the authors solicited the development of a sonographic tool for clinical practice to study regional perfusion in preterm babies.

Conversely, fetal magnetocardiography (fMCG) is a well-established diagnostic tool for fetal surveillance. Two original research papers describe the findings of studies performed with fMCG to estimate the development of the autonomic nervous system. It is known that, around 32 weeks of gestation, four fetal behavioral states (quiet and active sleep and awakeness) can be identified by combining fetal heart rate variability (fHRV) and fetal movements. Brändle et al. (2015) confirmed these results in 55 fetuses and also showed that only quiescence and active awakeness can be found in fetuses younger than 32 weeks, respectively in the 58.5 and 41.5% of cases. Therefore, fHRV parameters can differentiate fetal behavioral states at different ages and can show the neurovegetative modulation of each state, thus offering new insights into the vegetative development *in utero*. On the other hand, Hoyer et al. (2014) proved that the use of the fMCG based fetal Autonomic Brain Age Score (fABAS) may help estimating the fetal autonomic brain age, suggesting that the establishment of a fABAS score normogram is needed.

To understand the normal functional development of the fetal brain, periods of developmental vulnerability should be identified to assess the function of the fetal nervous system and open a window on novel prenatal diagnostics and prognostics. MRI has become increasingly feasible and clinically important in fetal brain studies due to its higher tissue resolution and better visualization of normal and pathological development of macroscopic anatomy or white matter microstructure and connections. Jakab et al. (2014) employed resting-state MRI (rsMRI) in 32 fetuses with no detectable morphological abnormalities to investigate the developmental changes in the functional connectivity architecture. They demonstrated that the short-range and interhemispheric connections show a sigmoid development peaking at around 26–29 gestational weeks, whereas long-range connections do not show any peak. They also observed an increasing region-specific functional connectivity from 24 to 28 gestational weeks, starting with the occipital and ending with the parietal cortex. Schöpf et al. (2014) used the same method to investigate the relationship between eye movements and brain functional activity in seven fetuses. The emergence of the visuomotor system involves the relationship of intrinsic and extrinsic components supposed to shape the subsequent development of perception. Schöpf and colleagues showed that spontaneous fetal eye movements are linked to the simultaneous networks in visual and frontal brain regions, demonstrating that the preparation of the human visuomotor system links visual and motor areas already *in utero*.

Despite the advancements in monitoring techniques and perinatal care, clinicians still have limited means to predict neurodevelopmental outcomes and plan early intervention. In their opinion article, Giampietri et al. (2015) offer a succinct and clear overview of the non-invasive techniques actually employed. The authors focused on the neonatal application of magnetic resonance imaging (MRI), claiming that, although its low predictive power, advanced MRI techniques such as diffusion tensor imaging (DTI) or spectrography could be useful in anticipating the diagnosis of brain damage and should become part of standard clinical care. The recent advances in diffusion MRI (dMRI) have also a great potential for a better understanding of neuronal connectivity impairments in preterm babies, as brilliantly exposed by Dudink et al. (2015) in their review article, or in revealing structural connectivity changes in infants and toddlers with autism spectrum disorder (ASD), as discussed by Conti et al. (2015) in their review article, suggesting a shift from hyper- to hypo-connectivity at around 3 years of age.

The development of functional connectivity patterns from infancy to childhood were studied also by Berchicci et al. (2015), who investigated the intrahemispheric properties of the sensorimotor system from 3 to 60 months of life using a prototypal magnetoencephalographic (MEG) system. In line with prior findings on the development of the adult fronto-parietal network for adaptive online task control, which involves both segregation and integration, the authors showed that this network, which provides a neurophysiological basis for the action-perception coupling, evolved with age from a more random to an adult small-world organization, more efficient for both local and global information processing. Another study regarding the development of the infant motor and motivation systems was conducted by Moon et al. (2015), who designed a contingent sucking preference study to test neonatal motivation to the mother or an unfamiliar female. Although difficult to demonstrate, electrophysiological studies showed that newborns use prenatal experiences and the motivational system to produce responses to familiar sounds. The authors demonstrated a weak neonate's contingent sucking response to the maternal voice, which was ascribed to insufficient neonates' motivation to alter their behavior, therefore pointing to the complementary value of electrophysiological and behavioral studies for very early development.

Finally, electroencephalography (EEG) is the most commonly used technique to non-invasively assess neonatal brain activity, but the main challenge in interpreting EEG signals is the quantitative characterization of the spontaneous “background activity” in sick neonates. Matic et al. (2015) applied multifactorial detrended fluctuation analysis (MF-DFA) to long-term EEG from 34 asphyxiated neonates to distinguish different grades of abnormality in EEG background activity, which could help monitoring the brain state changes occurring during long periods of time. The EEG from a normal developing neonate also presents specific characteristics. Koolen et al. (2014) used the interhemispheric synchrony (HIS) and the activation synchrony index (ASI) to analyze EEG traces from normal and abnormal neonates, and found these measures promising for diagnostic and clinical purposes. In particular, the ASI was able to correctly distinguish between normal and abnormal neonates in the 97% of cases.

## Conflict of interest statement

The authors declare that the research was conducted in the absence of any commercial or financial relationships that could be construed as a potential conflict of interest.
